# A Facile Synthesis and Anticancer Activity Evaluation of Spiro[Thiazolidinone-Isatin] Conjugates

**DOI:** 10.3797/scipharm.1109-14

**Published:** 2011-10-03

**Authors:** Danylo Kaminskyy, Dmytro Khyluk, Olexandr Vasylenko, Lucjusz Zaprutko, Roman Lesyk

**Affiliations:** 1 Department of Pharmaceutical, Organic and Bioorganic Chemistry, Danylo Halytsky Lviv National Medical University, Pekarska 69, 79010, Lviv, Ukraine; 2 Institute of Bioorganic Chemistry and Petrochemistry, National Academy of Science of Ukraine, Murmanska 1, 02094, Kyiv, Ukraine; 3 Department of Organic Chemistry, Poznan University of Medical Sciences, Grunwaldzka 6, 60-780, Poznań, Poland

**Keywords:** Spiro thiazolidinone isatin conjugates, 2,3,5-Trisubstituted 4-thiazolidinones, Anticancer activity, Spiro[indole-3, 2′-[1,3]thiazolidine]

## Abstract

The synthesis and evaluation of the anticancer activity of 3′-aryl-5′-arylidene-spiro[3*H*-indole-3,2′-thiazolidine]-2,4′(1*H*)-diones and spiro[3*H*-indole-3,2′-thi-azolidine]-2,4′(1*H*)-dione-3′-alkanoic acid esters were described. The structure of the compounds was determined by ^1^H and ^13^C NMR and their *in vitro* anticancer activity was tested in the National Cancer Institute. Among the tested compounds, (5′*Z*)-5′-(benzylidene)-3′-(4-chlorophenyl)spiro[3*H*-indole-3,2′-thia-zolidine]-2,4′(1*H*)-dione (**IIa**) and (5′*Z*)-3′-(4-chlorophenyl)-5′-[4-(1-methylethyl)-benzylidene]spiro[3*H*-indole-3,2′-thiazolidine]-2,4′(1H)-dione (**IIb**) were superior to other related compounds.

## Introduction

Design of new substances based on privileged scaffolds is one of the successful directions in drug discovery. According to this approach, the use of thiazolidinones (rhodanine, 2,4-thiazolidinone, hydantoin) and isatin (1*H*-indole-2,3-dione) gives access to series of compounds with a broad spectrum of biological activity. Traditionally, thiazolidinone derivatives are of great interest as sources of innovative drug candidates with antimicrobial, antiviral, antidiabetic effects etc. [1, 2]. Recently research of thiazolidinone’s pharmacological effects became interesting and promising for anticancer agents design [3, 4]. Broad spectrum of chemical modifications of the core heterocycles allowed to form diverse groups of thiazolidinone based derivatives [1, 3, 5, 6].

One of the above mentioned groups, 2,3-disubstituted 4-thiazolidinones [7], possesses the same spectrum of biological activity [8] including anticancer activity [9, 10]. On the other hand, isatin derivatives are also a well-known class of biological active compounds [11] and thus often used as the source of new drug candidates including anticancer agents [12]. The exploration of the isatin scaffold in combination with other molecular fragments is an effective approach for the design of drug-like substances [13]. The molecular mechanisms of their anticancer action is associated with the affinity to tyrosine kinase [14], cyclin-dependent kinases [15], carbonic anhydrase [16], inhibition of TNFα [17] etc.

**Fig. 1:**
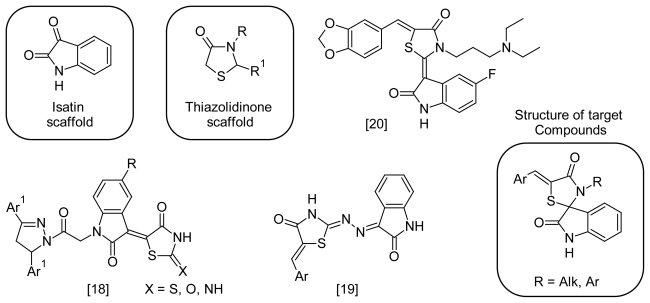
Structure of some isatin-thiazolidinone hybrid molecules with anticancer activity and structure of target compounds.

Combination of these two mentioned scaffolds in one molecule according seems to be a promising ‘hybrid pharmacophore’ approach to new anticancer agents. Some known examples of such hybrid molecules with anticancer activity are shown in figure 1 [18–20].

Dictated by the previous research results of thiazolidinone derivatives, the aim of the presented work was to synthesize new 2,3,5-trisubstituted 4-thiazolidinones bearig an isatin fragment and to investigate their anticancer activity.

## Results and Discussion

### Chemistry

The most convenient method for the synthesis of 2,3-disubstituted-4-thiazolidinones is the one-pot three-component reaction of a primary amine, an oxo-compound and a thiolic agent. The different reaction conditions, such as long term heating with a dehydrant, using an acylation agent or microwave assistant organic synthesis (MAOS) were described [21–28]. Using isatin or its derivatives in this reaction as oxo-compounds allowed to obtain spiro[indole-thiazolidinones] [24, 29]. Based on the above mentioned approach we have synthesized spirocompounds **I** in anhydrous benzene (Scheme 1).

Compounds **I** contain an active methylene group in position C5 of the core ring, which opens wide opportunities for their modification, taking into consideration the critical influence of the presence and the nature of the C5-position moieties on biological activity [1, 30]. The synthesis of 5-ylidene-4-thiazolidinones is realized in a Knoevenagel reaction but under different conditions. Commonly applied conditions (acetic acid and sodium acetate as catalyst) are not effective in 2-substituted-4-thiazolidinones because of the low reactivity of the methylene group in comparison with rhodanine (2-thioxo-4-thiazolidinone) or 2,4-thiazolidinedione derivatives [31, 32]. Therefore, the reaction was performed in *iso*-propanol with potassium *tert*-butylate as catalyst.

One of the facile methods of structure optimization is the introduction of amino acid residues into the molecules. Unfortunately, amino acids usage as amine component in the one-pot three component reaction (scheme 1) for the synthesis of compound with an aliphatic residue in position N3 of the core ring was not effective. For the synthesis of the target compounds **Ic, Id** the MAOS approach was used (Scheme 2).

The ^1^H NMR spectra of compounds **I** show two doublets at ~3.80–4.20 ppm of the thiazolidine methylene group. The chemical shift of the methylidene group of the 5-arylidenederivatives (**II**) is insignificantly displaced in the weak magnetic field ~7.40–8.00 ppm, and clearly indicates that only the *Z*-isomers were obtained [1, 30].

### Biological activity

Newly synthesized compounds were selected by the National Cancer Institute (NCI) Developmental Therapeutic Program (www.dtp.nci.nih.gov) for the *in vitro* cell line screening to investigate their anticancer activity. Anticancer assays were performed according to the US NCI protocol [33–36]. The compounds were first evaluated at one dose primary anticancer assay towards 60 cancer lines (concentration 10^−5^ M). The human tumor cell lines were derived from nine different cancer types: leukemia, melanoma, lung, colon, CNS, ovarian, renal, prostate and breast cancers. In the screening protocol, each cell line was inoculated and pre-incubated for 24–48 h on a microtiter plate. Test agents were then added and the culture was incubated for further 48 h. End point determinations were made with a protein binding dye, sulforhodamine B (SRB). Results for each test agent were reported as the percent growth of the treated cells when compared to the untreated control cells. The preliminary screening results are shown in Table 1.

The tested compounds showed different levels of anticancer activity and possessed the significant specific influence on some cancer cell lines. This activity pattern appeared probably due to distinctive molecular mechanisms of action of the mentioned substances. Among the tested cancer cell lines, the leukemia panel is the most susceptible to **IIa**, **IIb** and **IIc** influence. This action probably may be considered as a 4-thiazolidinones group feature [3, 30]. Compounds **Ic**, **Id**, **IIe** and **IIf**, do not possess significant anticancer action and therefore probably are not perspective substances for anticancer agents design. Nevertheless, compounds **IIa**, **IIb**, **IIc** and **Ic** specifically restrained the growth of UO-31 cancer line (renal cancer) in comparison with other cell lines. This specific pattern was observed under the action of related thiazolidinone derivatives [4, 30, 37, 38]. More prominent antiproliferative effect of **IIa**, **IIc**, **IIe**, **IIf** and **Ic** on SNB-75 cell line (CNS cancer) was detected as well. Elongation of the hydrocarbon chain in position N3 leaded to a decreasing activity level (comparison of activity pattern of **Ic** and **Id**).

Two compounds (**IIa** and **IIb**) were tested in a five concentrations assay. A 48 h continuous drug exposure protocol was used with a SRB protein assay to estimate the cell viability and growth. Results (Tables 2 and 3) are expressed as pGI_50_, pTGI, pLC_50_. (GI_50_ – molar concentration of the compound that inhibits 50% net cell growth; TGI – molar concentration of the compound leading to total inhibition of cell growth; LC_50_ – molar concentration of the compound leading to 50% net cell death). Values were calculated for each of these parameters if the level of activity was reached; however, if the effect was not reached or was exceeded, the value was expressed as more or less than the maximum or minimum concentration tested. Furthermore, mean graph midpoints (MG_MID) were calculated for each of the parameters, giving an average activity parameter over all cell lines for each compound. For the calculation of the MG_MID, insensitive cell lines were included with the highest concentration tested.

The selectivity index (SI) obtained by dividing the full panel MG-MID (μM) of the compound **IIa** and **IIb** by their individual sub-panel MG-MID (μM) was considered as a measure of a compound’s selectivity. Ratios between 3 and 6 refer to moderate selectivity, ratios greater than 6 indicate high selectivity toward the corresponding cell line, while compounds not meeting either of these criteria are rated nonselective [39, 40]. The selectivity pattern showed that compounds possess the moderate level of selectivity to Leukemia panel at both the GI50 and TGI levels (Table 4).

### COMPARE Analysis

NCI web-resources allow to compare selectivity patterns (mean graph fingerprints according DTP protocol) of the tested compounds with standard anticancer agents, and NCI active synthetic compounds and natural extracts, which are present in public available databases. Such analysis is based on the comparing the patterns of differential growth inhibition for cultured cell lines and can potentially gain insight into the mechanism of the cytotoxic action. If the data pattern correlates well with that of the compounds belonging to a standard agent database (Pearson’s correlation coefficient >0.6), the compound of interest may have the same mechanism of action. On the other hand, if the activity pattern does not correlate with any standard agent, it is possible that the compound has a novel mechanism of action. Standard COMPARE analyses (http://dtp.nci.nih.gov/docs/compare/compare.html) [41] were performed at GI_50_ level for **IIa** and **IIb**. Obtained correlation coefficients (r) didn’t allow distinguishing cytotoxicity mechanism of tested compounds with high probability. Nevertheless insignificant correlations with tamoxifen (NSC180973, r = 0.545), caracemide (NSC253272, r = 0.528), as well as fluorodopan (NSC73754, r = 0.533) for compound **IIa** and fluorodopan (NSC73754, r = 0.501) for **IIb** were detected. Interesting, that other 4-azolidinone derivatives also have significant value of correlation coefficients to the above mentioned substance [37, 38].

## Experimental

### Chemistry

Melting points were measured in open capillary tubes on a BUCHI B-545 melting point apparatus and are uncorrected. The elemental analyses (C, H, N) were performed using the Perkin–Elmer 2400 CHN analyzer and were within 0.4% of the theoretical values. The ^1^H- and ^13^C-NMR spectra were recorded on Varian Gemini 400 MHz or Bruker 125 MHz for frequencies 100 MHz in DMSO-*d*6 using tetramethylsilane as an internal standard. Chemical shifts are reported in ppm units with use of d scale.

#### General method for preparation of spiro[3H-indole-3,2′-thiazolidine]-3′-aryl-2.4′(1H)-diones (I)

A mixture of isatin (10 mmol), the appropriate amine (10 mmol) and acetic acid (1 ml) in anhydrous benzene was refluxed for 2 hours. Thioglycolic acid (20 mmol) was added and the reaction mixture was refluxed with a Dean-Stark apparatus for 8 hours. After cooling, the reaction mixture was concentrated and added to a sat. NaHCO_3_ solution. The obtained product was filtered and recrystallized.

##### 3′-(4-Chlorophenyl)-spiro[3*H*-indole-3,2′-thiazolidine]-2,4′(1*H*)-dione (3′-(4-Chlorophenyl)-4′*H*-spiro[indole-3,2′-[1,3]thiazolidine]-2,4′(1*H*)-dione, **Ia**)

Yield: 75%, mp 170–173°C (MeOH). ^1^H NMR (400 MHz, DMSO-*d_6_*): 4.02 (d, *J* = 15.6 Hz, 1H, CH_2_), 4.17 (d, *J* = 15.6 Hz, 1H, CH_2_), 6.78 (d, *J* = 7.7 Hz, 1H, isatin), 6.97–7.06 (m, 3H, arom.), 7.24 (t, *J* = 7.6 Hz, 1H, isatin), 7.38 (d, *J* = 8.5 Hz, 2H, arom.), 7.53 (d, *J* = 7.4, Hz, 1H, isatin), 10.84 (s, 1H, NH). ^13^C NMR (100 MHz, DMSO-*d_6_*,): 176.5, 172.31, 142.0, 135.6, 133.3, 131.78, 130.9, 129.9, 126.9, 125.0, 123.3, 111.3, 69.9, 32.8. Anal. Calcd for C_16_H_11_ClN_2_O_2_S, % C, 58.10; H, 3.35; N, 8.47. Found, %: C, 58.30; H, 3.55; N, 8.60.

##### 3′-(4-Hydroxyphenyl)-spiro[3*H*-indole-3,2′-thiazolidine]-2,4′(1*H*)-dione (3′-(4-Hydroxyphenyl)-4′*H*-spiro[indole-3,2′-[1,3]thiazolidine]-2,4′(1*H*)-dione, **Ib**)

Yield: 62%, mp >230°C (DMF:EtOH, 1:1). ^1^H NMR (400 MHz, DMSO-*d_6_*): 3.95 (d, *J* = 15.5 Hz, 1H, CH_2_), 4.11 (d, *J* = 15.5 Hz, 1H, CH_2_), 6.62 (d, *J* = 8.6 Hz, 2H, arom.), 6.73 (d, *J* = 7.7 Hz, 1H, isatin), 6.81 (d, *J* = 8.5 Hz, 2H, arom.), 7.01 (t, *J* = 7.5 Hz, 1H, isatin), 7.22 (t, *J* = 7.6 Hz, 1H, isatin), 7.53 (d, *J* = 7.4 Hz, 1H, isatin), 9.64 (s, 1H, OH), 10.71 (s, 1H, NH). ^13^C NMR (100 MHz, DMSO-*d_6_*,): 176.8, 172.3, 157.7, 142.1, 131.5, 130.2, 127.6, 127.0, 125.6, 123.1, 116.2, 111.0, 70.1, 32.7. Anal. Calcd for C_16_H_12_N_2_O_3_S, % C, 61.53; H, 3.87; N, 8.97. Found, %: C, 61.70; H, 4.00; N, 9.10.

### General method for the preparation of 3′-aryl-5-arylidene- spiro[3H-indole-3,2′-thiazolidine]-2,4′(1H)-diones (II)

A mixture of compound **I** (5 mmol), the appropriate aldehyde (5.5 mmol) and potassium *tert*-butylate (7.5 mmol) in *iso*-propanol was refluxed for 3 hours. The reaction mixture was cooled and acetic acid (1 ml) was added. The precipitate was filtered and recrystallized from an appropriate solvent.

#### (5′*Z*)-3′-(4-Chlorophenyl)-5′-(phenylmethylidene)spiro[3H-indole-3,2′-thiazolidine]- 2,4′(1H)-dione((5′Z)-5′-Benzylidene-3′-(4-chlorophenyl)-4′H-spiro[indole-3,2′-[1,3]thiazolidine]- 2,4′(1H)-dione, **IIa**)

Yield 63%, mp >230°C (*i*-PrOH). ^1^H NMR (400 MHz, DMSO-*d_6_*): 6.81 (d, *J* = 7.7 Hz, 1H, isatin), 7.03 (t, *J* = 7.6 Hz, 1H, isatin), 7.12 (d, *J* = 8.8 Hz, 2H, arom.), 7.26 (t, *J* = 6.9, Hz, 1H, isatin), 7.38–7.57 (m, 7H, arom.), 7.64 (s, 1H, PhCH=), 7.71 (d, *J* = 7.5, Hz, 1H, isatin), 11.01 (s, 1H, NH). ^13^C NMR (100 MHz, DMSO-*d_6_*,): 174.3, 166.6, 142.3, 135.1, 134.7, 133.8, 132.3, 130.8, 130.0, 129.9, 129.6, 129.5, 127.3, 127.1, 124.6, 124.1, 123.7, 111.5, 69.67. Anal. Calcd for C_23_H_15_ClN_2_O_2_S, % C, 65.95; H, 3.61; N, 6.69. Found, %: C, 66.10; H, 3.80; N, 6.95.

#### (5′*Z*)-3′-(4-Chlorophenyl)-5′-[[4-(1-methylethyl)phenyl]methylene]spiro[3*H*-indole-3,2′-thiazolidine]-2,4′(1*H*)-dione((5′Z)-3′-(4-Chlorophenyl)-5′-[4-(propan-2-yl)benzylidene]-4′*H*-spiro[indole-3,2′-[1,3]thiazolidine]-2,4′(1*H*)-dione, **IIb**)

Yield 68%, mp >230°C (AcOH). ^1^H NMR (400 MHz, DMSO-*d_6_*): 1.20 (d, *J* = 6.9 Hz, 6H, 2*CH_3_), 2.92 (m, 1H, CH), 6.83 (d, *J* = 7.8 Hz, 1H, isatin), 7.07 (t, *J* = 7.5 Hz, 1H, isatin), 7.13 (d, *J* = 8.5 Hz, 2H, arom.), 7.30 (t, *J* = 7.6 Hz, 1H, isatin), 7.36 (d, *J* = 8.8 Hz, 2H, arom.), 7.44 (d, *J* = 8.5 Hz, 2H, arom.), 7.48 (d, *J* = 8.0 Hz, 2H, arom.), 7.63 (s, 1H, ArCH=), 7.71 (d, *J* = 7.4 Hz, 1H, isatin), 11.03 (s, 1H, NH). ^13^C NMR (100 MHz, DMSO-*d_6_*,): 174.4, 166.8, 150.2, 142.3, 135.2, 133.8, 132.3, 132.2, 130.8, 130.0, 129.9, 127.5, 127.2, 127.1, 124.2, 123.6, 123.5, 111.5, 69.6, 33.8, 24.1. Anal. Calcd for C_26_H_21_ClN_2_O_2_S, % C, 67.74; H, 4.59; N, 6.08. Found, %: C, 67.50; H, 4.75; N, 6.21.

#### (5′*Z*)-5′-[[4-(Dimethylamino)phenyl]methylene]-3′-(4-hydroxyphenyl)spiro[3*H*-indole-3,2′-thiazolidine]-2,4′(1*H*)-dione((5′*Z*)-5′-[4-(Dimethylamino)benzylidene]-3′-(4-hydroxyphenyl)-4′*H*-spiro[indole-3,2′-[1,3]thiazolidine]-2,4′(1*H*)-dione, **IIc**)

Yield 59%, mp >230°C (DMF:EtOH, 1:1). ^1^H NMR (400 MHz, DMSO-*d_6_*): 2.97 (s, 6H, 2*CH_3_), 6.65 (d, *J* = 8.6 Hz, 2H, arom.), 6.76–6.79 (m, 3H, arom., isatin), 6.85 (d, *J* = 8.6 Hz, 2H, arom.), 7.06 (t, *J* = 7.5 Hz, 1H, isatin), 7.27 (t, *J* = 7.7 Hz, 1H, isatin), 7.37 (d, *J* = 8.6 Hz, 2H, arom.), 7.47 (s, 1H, ArCH=), 7.64 (d, *J* = 7.4, Hz, 1H, isatin), 9.69 (s, 1H, OH), 10.86 (s, 1H, NH). ^13^C NMR (100 MHz, DMSO-*d_6_*,): 174.9, 167.3, 157.8, 150.8, 142.3, 131.7, 131.4, 130.6, 127.3, 127.0, 125.4, 123.4, 122.2, 118.2, 118.1, 116.2, 112.5, 111.2, 69.7. Anal. Calcd for C_25_H_21_N_3_O_3_S, % C, 67.70; H, 4.77; N, 9.47. Found, %: C, 68.00; H, 5.00; N, 9.63.

#### (5′*Z*)-3′-(4-Hydroxyphenyl)-5′-(3-phenyl-2-propenylidene)spiro[3*H*-indole-3,2′-thiazolidine]-2,4′(1*H*)-dione((5′*Z*)-3′-(4-Hydroxyphenyl)-5′-(3-phenylprop-2-en-1-ylidene)-4′*H*-spiro[indole-3,2′-[1,3]thiazolidine]-2,4′(1*H*)-dione, **IId**)

Yield 72%, mp >230°C (AcOH). ^1^H NMR (400 MHz, DMSO-*d_6_*): 6.57–6.70, 6.74–6.82, 6.84–7.13, 7.17–7.41, 7.48–7.52 (5*m, 16H, PhCHCHCH, isatin, arom.), 9.68 (s, 1H, OH), 10.87 (s, 1H, NH). ^3^C NMR (100 MHz, DMSO-*d_6_*,): 174.6, 166.3, 157.9, 142.3, 139.2, 136.7,131.9, 130.4, 130.2, 129.8, 129.5, 129.4, 129.3, 127.6, 127.2, 127.1, 126.3, 125.0, 1234.4, 123.4, 116.2, 111.3, 69.7. Anal. Calcd for C_25_H_21_N_3_O_3_S, % C, 67.70; H, 4.77; N, 9.47. Found, %: C, 68.00; H, 5.00; N, 9.63.

#### 4-[(*Z*)-[1,2-Dihydro-3′-(4-hydroxyphenyl)-2,4′-dioxospiro[3*H*-indole-3,2′-thiazolidin]- 5′-ylidene]methyl]-2-methoxy-benzeneacetic acid((4-′(*Z*)-[3′-(4-Hydroxyphenyl)-2,4′-dioxo-1,2-dihydro-5′*H*-spiro[indole-3,2′-[1,3]thiazolidin]-5′-ylidene]methyl}-2-methoxyphenyl)acetic acid, **IIe**)

Yield 64%, mp >230°C (MeOH). ^1^H NMR (400 MHz, DMSO-*d_6_*): 3.86 (s, 1H, CH_3_O), 4.20 (s, 2H, CH_2_O), 6.60–6.70, 6.78–6.95, 7.00–7.12, 7.17–7.31, 7.50–7.66 (5*m, 12H, arom., isatin), 9.81 (s, 1H, OH), 10.68 (s, 1H, NH). ^13^C NMR (100 MHz, DMSO-*d_6_*,): 179.2, 176.1, 167.3, 144.1, 143.2, 139.3 135.5, 135.1, 133.8, 133.3, 131.8, 130.3, 129.9, 129.2, 128.9, 127.5, 127.0, 125.6, 124.8, 124.1, 111.1, 70.10, 61.3, 54.63. Anal. Calcd for C_26_H_20_N_2_O_7_S, % C, 61.90; H, 4.00; N, 5.55. Found, %: C, 62.10; H, 4.21; N, 5.73.

#### (5′*Z*)-5′-[(4-Bromophenyl)methylene]-3′-(4-hydroxyphenyl)spiro[3*H*-indole-3,2′-thiazolidine]-2,4′(1*H*)-dione((5′*Z*)-5′-(4-Bromobenzylidene)-3′-(4-hydroxyphenyl)-4′*H*-spiro[indole-3,2′-[1,3]thiazolidine]-2,4′(1*H*)-dione, **IIf**)

Yield 68%, mp >230°C (AcOH). ^1^H NMR (400 MHz, DMSO-*d_6_*): 6.79 (d, *J* = 7.7 Hz, 1H, izatin), 7.02 (t, *J* = 7.6 Hz, 1H, isatin), 7.21 (d, *J* = 8.4 Hz, 2H, arom.), 7.28 (t, *J* = 7.6, Hz, 1H, isatin), 7.32 (d, *J* = 8.8 Hz, 2H, arom.), 7.40 (d, *J* = 8.8 Hz, 2H, arom.), 7.46 (d, *J* = 8.0 Hz, 2H, arom.), 7.64 (s, 1H, ArCH=), 7.71 (d, *J* = 7.5 Hz, 1H, isatin), 9.81 (s, 1H, OH), 10.79 (s, 1H, NH). ^3^C NMR (100 MHz, DMSO-*d_6_*,): 174.6, 167.2, 142.1, 135.9, 135.1, 134.2, 133.6, 132.1 131.6, 130.3, 129.6, 129.4, 128.0, 127.8, 124.4, 123.9, 123.5, 111.4, 69.8. Anal. Calcd for C_23_H_15_BrN_2_O_3_S, % C, 57.63; H, 3.15; N, 5.84. Found, %: C, 57.86; H, 2.95; N, 5.58.

### General method for preparation of spiro[3H-indole-3,2′-thiazolidine]-2,4′(1H)-dione-3′-alkanoic acids

A mixture of amino acid ester hydrochloride (4 mmol), isatin (8 mmol), mercaptoacetic acid (12 mmol), triethylamine or Huning base (4 mmol) and molecular sieves (4 Å, 0.10 g) in ethanol (10 mL) was irradiated (Plazmatronica RM 800) with microwaves (power 100 W) at 100 °C for 30 min (three 10 min cycles). After cooling, the reaction mixture was diluted with AcOEt (50 mL), sequentially washed with sat. NaHCO_3_, water, dried (MgSO_4_) and the solvent was removed *in vacuo*. The obtained product was purified by column chromate-graphy (silica gel 60–230 meg, hexane:AcOEt, 1:1) and crystallized from EtOH.

#### 1,2-Dihydro-2,4′-dioxospiro[3*H*-indole-3,2′-thiazolidine]-3′-acetic acid ethyl ester (Ethyl (2,4′-dioxo-1,2-dihydro-3′*H*-spiro[indole-3,2′-[1,3]thiazolidin]-3′-yl)acetate, **Ic**)

Yield 68%, mp 131–133°C (EtOH). ^1^H NMR (400 MHz, DMSO-*d_6_*): 1.07 (t, *J* = 7.2 Hz, 3H, CH_3_), 3.39 (m, 2H, CH_2_CH_3_), 3.96–3.99 (m, 4H, 2*CH_2_), 6.92 (d, *J* = 7.8 Hz, 1H, isatin), 7.07 (t, *J* = 6.6 Hz, 1H, isatin), 7.33 (m, 2H, isatin), 10.83 (s, 1H, NH). ^13^C NMR (100 MHz, DMSO-*d_6_*,): 175.5, 171.8, 167.5, 142.4, 131.6, 126.7, 123.3, 122.5, 110.9, 68.3, 60.9, 43.8, 32.0, 13.9. Anal. Calcd for C_14_H_14_N_2_O_4_S, % C, 54.89; H, 4.61; N, 9.14. Found, %: C, 55.03; H, 4.86; N, 9.08.

#### 1,2-Dihydro-2,4′-dioxospiro[3*H*-indole-3,2′-thiazolidine]-3′-propanoic acid methyl ester (Methyl 3-(2,4′-dioxo-1,2-dihydro-3′*H*-spiro[indole-3,2′-[1,3]thiazolidin]-3′-yl)propanoate, **Id**)

Yield 75%, mp 114–116°C (EtOH). ^1^H NMR (400 MHz, DMSO-*d_6_*): 2.33–2.40 (m, 2H, CH_2_), 3.14–3.22 (m, 2H, CH_2_), 3.53 (s, 3H, CH_3_), 3.71 (d, *J* = 15.2 Hz, 1H, 5-H), 3.93 (d, *J* = 15.2 Hz, 1H, 5-H), 6.93 (d, *J* = 7.7 Hz, 1H, isatin), 7.07 (t, *J* = 7.5 Hz, 1H, isatin), 7.30–7.36 (m, 2H, isatin), 10.73 (s, 1H, NH). ^13^C NMR (100 MHz, DMSO-*d_6_*,): 175.4, 172.3, 168.6, 142.7, 131.4, 126.4, 123.5, 122.6, 111.1, 68.5, 61.3, 43.5, 33.1, 31.2. Anal. Calcd for C_14_H_14_N_2_O_4_S, % C, 54.89; H, 4.61; N, 9.14. Found, %: C, 55.03; H, 4.86; N, 9.08.

### Cytotoxic activity against malignant human tumor cells

Primary anticancer *in vitro* assay was performed at human tumor cell lines panel derived from nine neoplastic diseases, in accordance with the protocol of the Drug Evaluation Branch, National Cancer Institute, Bethesda [33–36]. Tested compounds were added to the culture at a single concentration (10^−5^ M) and the cultures were incubated for 48 h. End point determinations were made with a protein binding dye, sulforhodamine B (SRB). Results for each tested compound were reported as the percent of growth of the treated cells when compared to the untreated control cells. The percentage growth was evaluated spectrophotometrically versus controls not treated with test agents.

The cytotoxic and/or growth inhibitory effects of the most active selected compounds were tested in vitro against the full panel of about 60 human tumor cell lines at 10-fold dilutions of five concentrations ranging from 10^−4^ to 10^−8^ M. A 48-h continuous drug exposure protocol was followed and an SRB protein assay was used to estimate cell viability or growth. Using the seven absorbance measurements [time zero, (Tz), control growth in the absence of drug, (C), and test growth in the presence of drug at the five concentration levels (Ti)], the percentage growth was calculated at each of the drug concentrations levels. Percentage growth inhibition was calculated as: [(Ti − Tz)/(C − Tz)] × 100 for concentrations for which Ti ≥ Tz, [(Ti − Tz)/Tz] × 100 for concentrations for which Ti < Tz. Three dose response parameters were calculated for each compound. Growth inhibition of 50% (GI_50_) was calculated from [(*Ti* − *Tz*)/(*C* − *Tz*)] × 100 = 50, which is the drug concentration resulting in a 50% lower net protein increase in the treated cells (measured by SRB staining) as compared to the net protein increase seen in the control cells. The drug concentration resulting in total growth inhibition (TGI) was calculated from *Ti* = *Tz*. The LC_50_ (concentration of drug resulting in a 50% reduction in the measured protein at the end of the drug treatment as compared to that at the beginning) indicating a net loss of cells following treatment was calculated from [(*Ti* − *Tz*)/ *Tz*] × 100 = −50. Values were calculated for each of these three parameters if the level of activity is reached; however, if the effect was not reached or was exceeded, the value for that parameter was expressed as greater or less than the maximum or minimum concentration tested. The log GI_50_, log TGI, log LC_50_ were then determined, defined as the mean of the log’s of the individual GI_50_, TGI, LC_50_ values. The lowest values are obtained with the most sensitive cell lines.

## Figures and Tables

**Sch. 1:**
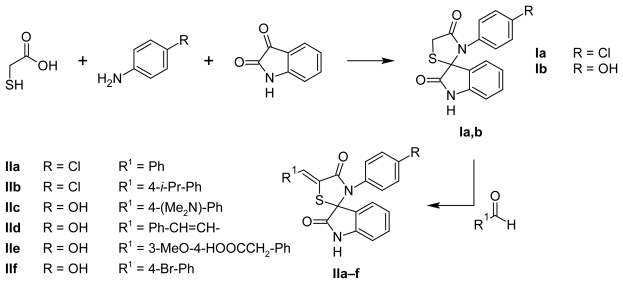
Spiro thiazolidinone-isatin conjugates synthesis.

**Sch. 2:**
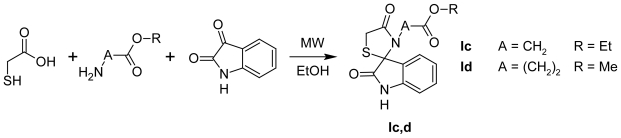
N3-alkyl substituted thiazolidinone-isatin conjugates synthesis.

**Tab. 1:** Cytotoxic activity of the tested compounds in concentration 10^−5^ M against 60 cancer cell lines

Cpd.	Mean growth, %	Range of growth, %	Most sensitive cell line growth, % (*cancer line*/type)
**Ic**	100.28	62.59–125.39	67.15 (*SR*/L); 62.59 (*UO-31*/RC)
**Id**	121.87	76.53–170.75	76.53 (*SR*/L)
**IIa**	55.72	1.78–89.28	24.52 (*A549/ATCC*/NSCLC); 39.71 (*BT-49*/BC); 1.78 (*T-47D*/BC); 31.30 (*CCRF-CEM*/L); 20.25 (*HL-60(TB)*/L); 33.28 (*K-562*/L); 10.67 (*MOLT-4*/L); 1 1.84 (*RPMI-8226*/L); 10.62 (*SR*/L); 17.91 (*UO-31*/RC); 24.77 (*SNB-75*/CNSC)
**IIb**	67.91	22.46–108.67	48.57 (*MCF-7*/BC); 22.46 (*T-47D*/BC); 28.68 (*OVCAR-4*/OC); 37.30 (*CCRF-CEM*/L); 36.53 (*HL-60(TB)*/L); 39.61 (*K-562*/L); 29.99 (*MOLT-4*/L); 27.22 (*RPMI-8226*/L); 37.26 (*SR*/L); 32.01 (*CAKI-1*/RC); 29.22 (*UO-31*/RC)
**IIc**	88.37	45.30–164.67	46.27 (*HL-60(TB)*/L); 45.30 (*K-562*/L); 44.68 (*MOLT-4*/L); 39.40 (*UO-31*/RC)
**IIe**	106.94	86.98–123.24	86.98 (*SNB-75*/CNSC); 90.19 (*CAKI-1*/RC); 91.15 (*UO-31*/RC)
**IIf**	104.78	87.84–129.52	87.84 (*OVCAR-4*/OC); 89.17 (*SNB-75*/CNSC)

ColC…colon cancer; M…melanoma; NSCLC…non-small cell lung cancer; RC…renal cancer; CNSC…CNS cancer; L…leukemia; BC…breast cancer; PC…prostate cancer; OV…ovarian cancer.

**Tab. 2:** Cytotoxic activity of IIa & IIb *in vitro* full panel 60-cell line assay

Cpd.	pGI_50_		pTGI		pLC_50_	

	Range^a^	MG_MID	Range^a^	MG_MID	Range^a^	MG_MID
IIa	5.71 to 4.27	5.27	5.18 to 4.03	4.17	4.28 to 4.10	4.01
	6.10 to 4.78^b^	5.25^b^	5.49 to 4.14^b^	4.31^b^	5.08 to 4.34^b^	4.05^b^
IIb	5.82 to 4.31	4.66	–	4.00	–	4.00
	6.55 to 4.13^b^	4.94^b^	–	4.02^b^	–	4.00^b^

a values < 4.00 were excluded;

b repeat assay.

**Tab. 3:** Cytotoxicity of the studied compounds against individual tumor cell lines

Cpd.	Cancer type	Most sensitive cell lines	pGI_50_	pTGI	pLC_50_
**IIa**	Leukemia	CCRF-CEM	5.64	5.20	< 4.00
		HL-60(TB)	5.90	5.49	5.08
		K-562	5.65	5.13	< 4.00
		MOLT-4	5.76	5.37	4.67
		RPMI-8226	5.74	5.28	< 4.00
		SR	5.70	5.17	< 4.00
	Non-small cell lung cancer	NCI-H322M	5.92	< 4.00	< 4.00
	Colon cancer	COLO 0205	5.73	5.36	4.99
	Melanoma	MALME-3M	6.10	5.30	< 4.00
	Renal cancer	TK-10	5.97	4.63	< 4.00
	Breast cancer	BT-549	5.83	4.52	< 4.00

**IIb**	Leukemia	K-562	5.59	< 4.00	< 4.00
		MOLT-4	5.56	< 4.00	< 4.00
	CNS cancer	SNB-75	5.72	< 4.00	< 4.00
	Renal cancer	CAKI-1	6.55	< 4.00	< 4.00
	Breast cancer	T-47D	5.63	< 4.00	< 4.00

**Tab. 4:** Anticancer selectivity pattern of the most active compounds at the GI_50_ (μM) and TGI (μM) levels.

Cancer type	GI_50,_	μM^a^	SI(GI_50_)^a^		TGI, μM^a^		SI(TGI)^a^	

	IIa	IIb	IIa	IIb	IIa	IIb	IIa	IIb
Leukemia	1.89	3.73	2.40	8,00	5.35	>100	12.38	0.99
	2.97	3.61	2.31	13.6	15.30	>100	5.14	1.00
NSC lung cancer	3.65	49.77	1.24	0.60	90.59	>100	0.76	0.99
	10.49	52.07	0.66	0.95	87.02	>100	0.89	1.00
Colon cancer	3.57	19.74	1.27	1.51	65.19	>100	1.05	0.99
	4.08	34.32	1.69	1.43	66.32	>100	1.16	1.00
CNS cancer	5.42	42.23	0.84	0.71	89.98	>100	0.76	0.99
	7.31	75.70	0.94	0.65	97.50	>100	0.79	1.00
Melanoma	3.74	15.07	1.21	2.00	23.33	>100	2.94	0.99
	8.26	53.31	0.83	0.92	68.20	>100	1.13	1.00
Ovarian cancer	5.97	46.95	0.76	0.64	>100	>100	0.69	0.99
	7.07	84.65	0.97	0.58	87.14	>100	0.88	1.00
Renal cancer	3.79	17.32	1.20	1.72	71.53	88.52	0.96	1.12
	4.43	40.00	1.55	1.23	80.38	>100	0.96	1.00
Prostate cancer	9.63	52.25	0.47	0.57	92.90	>100	0.74	0.99
	8.26	53.43	0.83	0.92	>100	>100	0.77	1.00
Breast cancer	3.47	21.47	1.31	1.39	77.55	>100	0.88	0.99
	5.48	45.70	1.25	1.07	92.35	>100	0.83	1.00

a repeat assay.
